# Liparthroplasty for Thumb Carpometacarpal Joint Osteoarthritis: A Case Series with Median 5 Years of Follow-Up

**DOI:** 10.3390/jcm11216411

**Published:** 2022-10-29

**Authors:** Matthias Holzbauer, Manfred Schmidt, Julian Alexander Mihalic, Dominik Duscher, Stefan Mathias Froschauer

**Affiliations:** 1 Department for Orthopedics and Traumatology, Med Campus III, Kepler University Hospital, Krankenhausstrasse 9, 4020 Linz, Austria; 2 Faculty of Medicine, Johannes Kepler University Linz, Altenbergerstraße 69, 4020 Linz, Austria; 3 Department of Plastic, Aesthetic, and Reconstructive Surgery, Med Campus III, Kepler University Hospital Linz, Krankenhausstrasse 3, 4020 Linz, Austria; 4 Department of Plastic, Reconstructive, Hand and Burn Surgery, BG-Trauma Center, Eberhard Karls University Tuebingen, Schnarrenbergstrasse 95, 72076 Tuebingen, Germany; 5 Diakonissen Clinic Linz, Weißenwolffstrasse 15, 4020 Linz, Austria

**Keywords:** arthroplasty, autologous fat transplantation, liparthroplasty, thumb carpometacarpal joint, osteoarthritis

## Abstract

Liparthroplasty has recently been discussed as a promising bridging therapy after failed conservative treatment options to postpone arthroplasty surgery of the thumb carpometacarpal joint as long as possible. The current study investigates the sustainability of this method in seven stage II and twenty-four stage III osteoarthritis patients (twenty-seven female and four male cases). Data were evaluated preinterventionally, six months postinterventionally, and two years postinterventionally, as well as a final follow-up assessment after median 5.1 years. We found a significant reduction of all postinterventional disabilities of the arm, shoulder, and hand (dash) scores and pain levels compared to the ones prior to liparthroplasty. Moreover, we even detected a reduction in both parameters within the postinterventional course, so that the DASH scores of our final investigation were significantly lower than the values after six months. Furthermore, 12 of our 31 cases demanded a surgical conversion due to recurrence of symptoms. A binary regression analysis found smokers to have 11 times higher odds for therapy failure, leading to surgical conversion. Seventeen out of nineteen patients in our final assessment stated that they were pleased with liparthroplasty. Due to favorable mid-term outcomes of 61% of the 31 initially treated patients, we recommend liparthroplasty as a reliable bridging therapy for preserving joint integrity as long as possible, especially in non-smoking patients.

## 1. Introduction

In patients presenting with symptomatic thumb carpometacarpal (CMC) joint osteoarthritis (OA), the most commonly applied treatment regimens start with non-surgical interventions. As in other joints affected with OA, nonsteroidal anti-inflammatory drugs are often used. However, especially non-selective agents imply a considerable risk for gastrointestinal and cardiovascular toxicity, as well as acute kidney injury [1]. As another more invasive step, corticosteroid and hyaluronic acid intra-articular injections are often performed [2]. While the former therapy implies a considerable short-term pain relief, chondrotoxity is known as an adverse effect that accelerates the progress of OA [3,4]. Moreover, a recent meta-analysis of these injection therapies in thumb CMC joint even concludes that there is no evidence that both intra-articular agents are superior to placebo [2]. Because these non-surgical options pursue no causal temporary symptom-relieving approach, patients often demand a long-term solution in case of recurrence of symptoms. Then, thumb CMC joint surgery is extensively performed even in early OA stages to causally address the site of pain origin. Currently, trapeziectomy with or without various ligament reconstruction and tendon interposition techniques still represents the surgical gold standard, while total joint replacement is becoming increasingly popular [5]. Because trapeziectomy is associated with a considerable loss of range of motion and strength [6] and the long-term performance of total arthroplasty is still under-investigated [7], there is a need for effective, non-surgical treatment to preserve the integrity of the thumb CMC joint as long as possible. In this regard, novel therapies, including intra-articular injection of platelet-rich plasma (PRP) [8] as well as adipose-derived mesenchymal stem cells in autologous lipoaspirated fat transplantation (AFT) [9], aim to avail the endogenous, immunosuppressive, and regenerative capability of autologous agents. In our previous study reporting our outcomes of AFT, i.e., liparthroplasty, we found a cumulative symptom remission rate of 58% after 2 years [10]. To investigate the mid-term performance and the sustainability of this novel therapy, our former study cohort was followed up for this study.

## 2. Materials and Methods

For the present study, we called in patients who were assessed in our previous study, including the 2 year postinterventional results [10], for another follow-up appointment. Between February 2017 and August 2018, patients with symptomatic, Eaton-Littler stage II to III thumb CMC joint OA were included in this study and treated with liparthroplasty.

Having exhausted prior conservative treatment, including thumb orthosis, antiphlogistic drugs, and hand therapy, was a prerequisite for inclusion. Any history of previous surgery or trauma involving Bennett’s fracture-dislocation and Rolando fracture, concomitant scapho-trapezio-trapezoid OA, rheumatoid arthritis, or earlier intra-articular injections were defined as exclusion criteria.

Our liparthroplasty technique was described in detail in our former publication [10]: After infiltrating 50 milliliters of tumescent solution, lipoaspirate was harvested in regional anesthesia from the lower abdominal region using a single umbilical incision. After the fat tissue was separated from fluids and oils using the decanting method, it was mechanically homogenized with two syringes. Next, 1 mL of lipoaspirate was injected into the thumb CMC joint under fluoroscopic guidance. Postinterventional regimen included a stabilizing thumb orthosis for 3 weeks.

The present study was approved by the responsible ethical review board (EK Nr: 1094/2018) and was performed in accordance with the World Medical Association (WMA) Declaration of Helsinki. Written informed consent to participate in this trial was obtained by all participants.

### 2.1. Parameters

Demographics of our study participants, including age at the time of surgery, sex, and affected side, were recorded. Moreover, patient’s body mass index (BMI), the affected side, the smoking status, comorbidities regarding arterial hypertension, and diabetes were noted.

At a baseline level before surgery, Disabilities of the Arm, Shoulder, and Hand (DASH) Scores and pain levels via a visual analogue scale (VAS) were assessed.

A 5-item Likert scale (1—“much better”, 2—“better”, 3—“unaltered status”, 4—“worse”, 5—“much worse”) for the assessment of change of clinical symptoms at 4, 8, and 16 weeks postinterventionally has already been reported in our previous study [10].

Six months after liparthroplasty, DASH Scores, pain via VAS, as well as the subjective change of grip strength compared to the preinterventional status using the above-described Likert-scale was assessed.

Two years postinterventionally and at a final follow-up appointment, DASH Scores, pain levels, subjective change of grip strength, and grip strength using a Jamar Dynamometer were evaluated. The final follow-up appointment also included a range of motion assessment, including radial and palmar abduction via the Radius-metacarpal angle method [11], and opposition was assessed as distance (in cm) between the thumb tip and the fifth metacarpophalangeal joint.

Patient’s satisfaction with liparthroplasty was evaluated at 6 months, 2 years, and final follow-up appointment, questioning if they would undergo the same procedure again.

Moreover, any occurring complications were recorded. In the case of recurrence of initial symptoms, thumb CMC prosthesis was offered as further escalation of therapeutic regimen. In any patient who opted for this surgical conversion, we reviewed the time between liparthroplasty and prosthesis implantation as well as clinical parameters (DASH, VAS, range of motion) in the medical records prior to arthroplasty surgery.

### 2.2. Statistical Methods

Demographics and clinical data were analyzed according to standard methods of descriptive statistics. Results are presented either as mean ± standard deviation or as median and interquartile range (IQR), respectively, depending on the result of the Shapiro–Wilk test for normal distribution. A Kaplan–Meier plot was used to visualize the cases which were converted to a thumb CMC joint prosthesis because of recurrence of symptoms. Vice versa, the curve displays the cumulative treatment response rate with surgical conversion as index event.

A binary logistic regression analysis was performed to detect any preinterventional predictors for a failed treatment response. First, a dummy variable for all cases with the need for surgical conversion was created. Following that, a univariate regression analysis was calculated using demographic data, comorbidities, smoking status, arthrosis stage as well as preinterventional DASH Scores and VAS levels. The odds ratios, including their 95% confidence intervals (CI), were given. If more parameters revealed a significant result, a multivariate regression model was calculated.

The Friedman test was applied to compare DASH Scores, pain levels, and subjective grip strength levels. If this test resulted in a significant result, pairwise comparisons were performed, and adjusted significant values according to the Dunn–Bonferroni post-hoc tests were given. For comparison of objective grip strength values, the paired T-Test or Wilcoxon-Test was used depending on the data’s normal distribution. Furthermore, satisfaction rates were analyzed using the Chi-Squared Test or the Fisher’s exact test in case any value was lower than 5. Comparison between clinical data of patients opting for surgical conversion prior prosthesis implantation and the data of patients attending the final follow-up appointment was performed either using the unpaired T-test or the Mann–Whitney U test depending on the data’s normal distribution.

The significance level was set at *p*-value < 0.05.

## 3. Results

13 left and 18 right thumbs (27 female and 4 male cases) with 7 stage II and 24 stage III OA were assessed. This cohort equaled the one of our previous study [10]. The median age was 57.7 (IQR: 10.0) years. Median BMI was 27 (7) kg/m^2^. We recorded 16 cases of smoking patients, 3 cases with diabetes, and 10 cases with arterial hypertension. Final follow-up assessment was performed median 5.1 (1.4) years postinterventionally.

**Figure 1:**
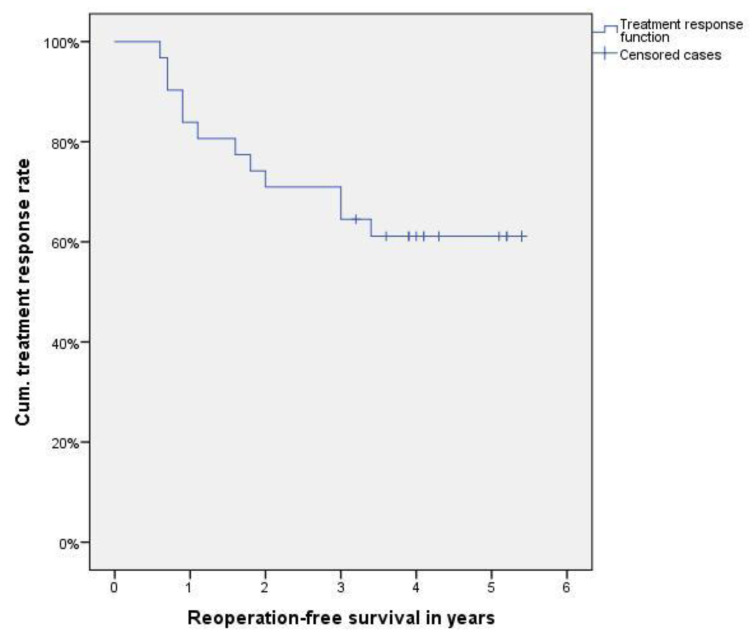
Kaplan–Meier plot indicating the cases requiring surgical conversion, resulting in a cumulative treatment response rate function.

Figure 1 visualizes the cases with the need for surgical conversion at the respective postinterventional time using a Kaplan–Meier plot.

Table 1 shows the odds ratios, including their 95% CI and significance values for the preinterventional parameters, predicting the need for surgical conversion. Thus, we found that smoking patients had 11 times higher odds for therapy failure requiring surgical conversion.

Table 2 provides the descriptive data of our clinical outcomes, as well as the results of the statistical comparison. Pairwise comparisons for DASH Scores showed a significant difference of the final assessment (*p* < 0.0001), 2 years (*p* < 0.0001), and 6 months (*p* = 0.03) values compared to the preinterventional ones. Moreover, the results of the final assessment were significantly lower than the ones 6 months postinterventionally (*p* = 0.007). Regarding pain, we found significantly lower levels at the final assessment (*p* < 0.0001), 2 years (*p* < 0.0001), and 6 months (*p* = 0.003) after liparthroplasty compared to the preinterventional values.

No postinterventional complications or adverse effects were detected.

Prior prosthesis implantation was performed at mean 1.6 ± 1.0 years, and the clinical data of 10/12 cases were available for a comparison to the ones of the 19 patients who attended the final follow-up appointment: Thus, patients requesting revision surgery had significantly worse function in all clinical parameters: DASH Scores (53 ± 18; *p* < 0.0001), pain levels via VAS was (7.5 (1.5); *p* = 0.0002), objective grip strength (3.9 ± 1.3; *p* = 0.001), radial abduction (55 (10)°; *p* = 0.003), palmar abduction (50 ± 7°; *p* = 0.001), and opposition (0 ± 1.8 cm; *p* = 0.013).

## 4. Discussion

The main outcome of this study is that the previously reported beneficial outcomes of liparthroplasty could still be detected after a median follow-up period of 5.1 years. In detail, the treatment response regarding pain relief and improvement of functional parameters even surpassed the values of our previous investigation [10].

Since the introduction of autologous lipoaspirated fat transplantation in the treatment of thumb CMC joint OA in a pilot study by Herold et al. in 2014 [12], this novel injection therapy using autologous fat tissue was investigated by several study groups. Haas et al. [13] found, in a non-randomized cohort study, that liparthroplasty surpasses the performance of corticosteroid injections after 3 months. Following this, the same author group reported the current largest case series regarding liparthroplasty, including 99 consecutive cases [14]. They found a continuous improvement of pain levels and Michigan Hand Outcomes Questionnaire scores during their assessments 2 weeks, 6 weeks, 3 months, 6 months, and 12 months postinterventionally. The only study comparing liparthroplasty (*n* = 9) to a trapeziectomy technique (*n* = 12), i.e., Lundborg’s resection arthroplasty, showed comparable functional results after 1.5 and 2 years of follow-up [15]. Moreover, they detected even a significant advantage in the liparthroplasty cohort regarding shorter time from surgery to absence of any pain. Despite these promising short-term results, all these publications discuss the factor sustainability of this novel technique in the limitations section [13,14,15]. In this regard, the literature provides a case series of 27 cases with a mean follow-up period of 3.8 years, reporting VAS values of 1.9 ± 1.7, DASH Scores of 30 ± 25, and a pinch grip strength of 5.1 ± 3.0 [16]. Besides a significant, postinterventional improvement of the first two parameters, the authors also report the need for surgical conversion of four patients. Recently, Herold et al. published a case series (*n* = 42) with the longest follow-up period, i.e., mean 4.9 ± 0.7 years [17]. The authors report that three patients out of the initial 50 consecutive patients received surgical conversion within the first postinterventional year. Compared to the previously mentioned study [16] and our outcomes, they present slightly higher pain levels at activity (4.0 ± 3.0), and DASH Scores (31 ± 22), while these results were still significantly lower than preinterventionaly.

Our study corroborates the current evidence—that liparthroplasty has the potential to achieve improvement of symptoms over 4 to 5 years—while other authors even report lower conversion rates between 7–14% [16,17]. Regarding invasive thumb CMC joint surgery, there is hardly any other joint with a higher range of various surgical approaches for OA described in the literature. Although total joint arthroplasty offered at our institution represents an increasingly popular method, currently any trapeziectomy with ligament reconstruction technique is the most commonly performed technique because of durably good outcomes over several decades in the literature [18]. Trost et al. [18] proved a very recent overview of currently available surgical methods and their respective functional advantages, complication profiles, and recovery times, concluding that there is still no hard evidence that one technique is superior to all others. Given the fact that symptoms of thumb CMC joint OA usually start in a patient’s late 50s [19], liparthroplasty gives the opportunity to delay joint-replacing surgery, involving longer recovery times after patients have reached retirement age [15].

While patients in our study are slightly younger than the ones of the previous studies regarding liparthroplasty (range of 59–66 years), the gender distribution is comparable (range of 66–88% female cases). However, the crucial factor in comparing the study population is the radiographic OA stage according to Eaton–Littler. While some studies involve stage I patients [13,14,16], other study cohorts also consist of stage IV patients [15,17]. Although the radiographic OA stage does principally not correlate with the severity of clinical symptoms [20], invasive treatment like liparthroplasty is only offered to patients with a certain level of suffering. Assuming the presence of a certain degree of symptoms, the radiographic stage indicates the stage of cartilage and joint degeneration. Thus, we could prove in our previous report that stage II patients have significantly lower postinterventional pain levels compared to stage III patients [10]. In this study, we found in the logistic regression analysis that smoking patients have 11 times higher odds of therapy failure and a need for surgical conversion.

While basis research provided proof of an anti-inflammatory and chondroprotective effects of adipose derived stem cells [9,21], the exact mode of action of liparthroplasty is still unclear. Intra-articular fat tissue my also involve beneficial mechanical properties in terms of a buffer effect cushioning force transduction, as well as favorable lubricant effects.

Recently, platelet-rich plasma (PRP) was reported as a novel approach of an intra-articular, autologous agent in thumb CMC joint OA treatment. Principally, its effects of reducing the inflammatory process and altering the cartilage metabolism are deemed similar to the ones of liparthroplasty. However, the evidence is still limited to publications reporting about results between 3 months and 1 year postinterventionally [22,23,24]. Compared to data regarding liparthroplasty, inhomogeneous cohorts involving stage I to IV stage OA patients showed similar DASH scores between 20 and 30 points, while pain levels were slightly higher, ranging between 2 and 6. However, PRP might have the advantage of the desired agent being able to be harvested by drawing blood from a peripheral vein without the requirement of an operating room, and previous studies have reported the need for a sequential injection of two sessions. Ultimately, this technique also must prove its sustainability in improvement of pain levels and functional parameters.

This study also contains certain limitations. First, objective grip strength measurements, as well as a range of motion data, are not available at all follow-up investigations. Although the subjective grip strength evaluation reflects a certain subjective opinion about the postinterventional strength development, the results allow no comparison to previously published data. Furthermore, the methodology lacks an adequate control group. Thus, the confounding factor of a placebo effect is not controlled in our results. However, the consistent outcomes in the postinterventional period suggest a minimal impact. Although the indication for surgical conversion was confirmed by significantly worse clinical parameters, these cases also represent a substantial loss of follow-up in the assessment of the mid-term performance of liparthroplasty. To determine the future role of liparthroplasty in the treatment regimen of thumb CMC joint OA further, larger studies are required to investigate the maximum duration of action, as well as the long-term performance compared to conventional surgical techniques.

## 5. Conclusions

This study shows that the investigated cohort of liparthroplasty patients showed a significant and sustainable pain reduction and improvement of functional parameters after a median follow-up period of 5.1 years. There was a continuous need for surgical conversion over the postinterventional period, so that 61% of 31 initially treated patients could be investigated in this study. Patients requesting surgical conversion presented with significantly worse functional scores, pain levels and range of motion data compared to patients attending our final follow-up appointment. A main outcome of this study is that smoking patients have a significantly higher risk for therapy failure. Thus, we conclude that liparthroplasty is a sustainable, reliable, and safe bridging therapy for preserving the thumb CMC joint as long as possible, which we do not recommend for smoking patients.

## Figures and Tables

**Table 1:** Odds ratios and significance level for surgical conversion.

	Odds Ratio (95% CI)	*p*-Value
DASH Score	0.998 (0.956; 1.041)	0.92
VAS for pain	1.207 (0.835; 1.746)	0.32
age	0.286 (0.854; 1.047)	0.29
sex	n/a	1.00
side	0.982 (0.227; 4.251)	0.98
stage	5.077 (0.528; 48.85)	0.16
smoker	10.83 (1.790; 65.55)	0.009
diabetes mellitus	n/a	1.0
arterial hypertension	1.083 (0.232; 5.061)	0.92

**Table 2:** Clinical outcomes including statistical comparison.

Parameters	Preop.	6 Months	2 Years	Final	*p*-Value
*n*	31	31	23	19	
DASH Scores	59 (26)	40 (43)	29 ± 19 (34)	22 ± 17	<0.0001
VAS for pain	7 (2)	4 (6)	2 (5)	1 (1)	<0.0001
subj. grip strength	n/a	0 (0)	0 (1)	0 (1)	0.26
obj. grip strength	-	-	6.4 ± 1.7	6.2 ± 2.1	0.21
radial abduction	-	-	-	60 (5)°	-
palmar abduction	-	-	-	60 (5)°	-
opposition	-	-	-	0 (0) cm	
satisfaction rate	n/a	21/31	16/23	17/19	0.20

## Data Availability

Data is contained within the article.
